# Different charged biopolymers induce α-synuclein to form fibrils with distinct structures

**DOI:** 10.1016/j.jbc.2024.107862

**Published:** 2024-10-05

**Authors:** Yuxuan Yao, Qinyue Zhao, Youqi Tao, Kaien Liu, Tianyi Cao, Zipeng Chen, Cong Liu, WeiDong Le, Jing Zhao, Dan Li, Wenyan Kang

**Affiliations:** 1 Bio-X Institutes, Key Laboratory for the Genetics of Developmental and Neuropsychiatric Disorders (Ministry of Education), Shanghai Jiao Tong University, Shanghai, China; 2 Zhangjiang Institute for Advanced Study, Shanghai Jiao Tong University, Shanghai, China; 3 Interdisciplinary Research Center on Biology and Chemistry, Shanghai Institute of Organic Chemistry, Chinese Academy of Sciences, Shanghai, China; 4 State Key Laboratory of Chemical Biology, Shanghai Institute of Organic Chemistry, Chinese Academy of Sciences, Shanghai, China; 5 School of Life Sciences and Biotechnology, Shanghai Jiao Tong University, Shanghai, China; 6 Chemistry and Biomedicine Innovation Center (ChemBIC), State Key Laboratory of Coordination Chemistry, School of Chemistry and Chemical Engineering, Nanjing University, Nanjing, China; 7 Liaoning Provincial Key Laboratory for Research on the Pathogenic Mechanisms of Neurological Diseases, The First Affiliated Hospital of Dalian Medical University, Dalian, China; 8 Department of Neurology and Institute of Neurology, Ruijin Hospital, Shanghai Jiao Tong University School of Medicine, Shanghai, China; 9 Department of Neurology, Ruijin Hainan Hospital, Shanghai Jiao Tong University, School of Medicine (Boao Research Hospital), Hainan, China

**Keywords:** α-synuclein, charged biopolymers, cryo-EM structure, amyloid, Parkinson's disease

## Abstract

The aggregation of α-synuclein (α-syn) into amyloid fibrils, a key process in the development of Parkinson's disease (PD) and other synucleinopathies, is influenced by a range of factors such as charged biopolymers, chaperones, and metabolites. However, the specific impacts of different biopolymers on α-syn fibril structure are not well understood. In our work, we found that different polyanions and polycations, such as polyphosphate (polyP), polyuridine (polyU), and polyamines (including putrescine, spermidine, and spermine), markedly altered the fibrillation kinetics of α-syn *in vitro*. Furthermore, the seeding assay revealed distinct cross-seeding capacities across different biopolymer-induced α-syn fibrils, suggesting the formation of structurally distinct strains under different conditions. Utilizing cryo-electron microscopy (cryo-EM), we further examined the detailed structural configuration of α-syn fibrils formed in the presence of these biopolymers. Notably, we found that while polyamines do not change the atomic structure of α-syn fibrils, polyU and polyP induce the formation of distinct amyloid fibrils, exhibiting a range of structural polymorphs. Our work offers valuable insights into how various charged biopolymers affect the aggregation process and the resultant structures of α-syn fibrils, thereby enhancing our understanding of the structural variations in α-syn fibrils across different pathological conditions.

Parkinson's disease (PD) is the most prevalent movement disorder and ranks second among neurodegenerative disorders. It is characterized by the abnormal accumulation of α-synuclein (α-syn) in Lewy bodies (LBs) and Lewy neurites (LNs) (1, 2, 3, 4). α-Syn, an inherently disordered protein, plays a role in synaptic vesicle trafficking under normal conditions (5, 6). However, in PD, α-syn self-assembles into amyloid fibrils, featuring a highly ordered cross-β-sheet structure (7, 8, 9, 10). α-Syn is composed of three regions: the N-terminal region, which is amphipathic and lysine-rich, interacts with membranes (11); the central non-amyloid component (NAC) region, shifts from a disordered structure in monomers to a β-sheet-rich structure in fibrils, which is vital in forming the fibril core (12); and the C-terminal region, rich in negatively charged amino acids, mediates the binding of α-syn fibrils to various receptors (13, 14).

Mounting evidence demonstrates that various factors, such as lipids, chaperones, protein binding partners, post-translational modifications (PTMs), and metabolites are enrolled in the process of pathological α-syn aggregation (15, 16, 17, 18, 19). Moreover, the inherently charged nature of α-syn allows it to interact with various charged biopolymers like heparin, nucleic acids, and polyamines, which further significantly affect its fibrillation kinetics (20, 21, 22). The prevalence of polycations and polyanions in cellular environments necessitates exploring how they impact α-syn fibrillation. Polyamines are abundant polycations of vital importance in mammalian cells (23, 24, 25). Additionally, early-onset PD is associated with mutations in the polyamine transporter ATP13A2, which implicates polyamine disturbance involved in α-syn aggregation and neurodegeneration in PD (26, 27, 28). As for polyanions, polyphosphate (polyP), a highly negatively charged biopolymer, is known to induce fibrillation in various amyloid proteins (29, 30). *In vitro* studies have shown that polyP, especially those with 60 to 70 phosphates, can trigger α-syn fibril formation by interacting with α-syn's positively charged “KTK” motifs (31). Similarly, nucleic acids, as biomolecular polyanions, significantly influence protein aggregation, as seen in Tau and amyotrophic lateral sclerosis (ALS)-related RNA binding proteins like TAR DNA-binding protein (TDP-43), fused in sarcoma (FUS), heterogeneous ribonucleoprotein1 (hnRNPA1), and others (32, 33, 34, 35). Nonetheless, the specific effects of these charged biopolymers on α-syn fibril structure are not well understood.

In this work, utilizing cryo-electron microscopy (cryo-EM), we discovered that different biopolymers, including polyamines, polyuridine (polyU), and polyP, present varying impacts on α-syn fibril formation. Specifically, polyamines alter the fibrillation kinetics without changing the overall fibril structure of α-syn. In contrast, polyU and polyP affect both the fibrillation kinetics and the final morphology of α-syn fibril, albeit in different manners. Our findings provide structural insights into the modulation mechanism of charged polymers on α-syn fibrillation.

## Results

### Polyamines modulated α-syn fibrillation without changing the fibril structure

We first sought to examine how various polyamines affect α-syn fibrillation. Thioflavin-T (ThT) fluorescence assay was used to monitor the kinetics of α-syn fibrillation in the presence and absence of three different polyamines, including putrescine, spermidine, and spermine. The assay was conducted under the fibrillation condition of 50 mM Tris-HCl buffer at pH 7.5, and 150 mM KCl with varying polyamine concentrations. No significant accelerating effects of polyamines in the α-syn fibrillation process were observed, except at higher concentrations (spermidine: 10 mM; spermine: 2–10 mM) where polyamines inhibited fibrillation (Fig. S1). It is further demonstrated by negative staining transmission electron microscopy (NS-TEM) images, which showed no obvious difference in the quantity of fibrils formed under different polyamine concentrations (Fig. S2). It could be explained that both polyamines and salt ions could interact with the C-terminal region of α-syn through electrostatic interaction. When compared with salt ions, polyvalent cations like polyamines do more than just neutralize. They can also improve local α-syn concentration, potentially promoting fibril formation. However, the involvement of salt might compete with polyamines for the binding to α-syn, thus disrupting the accelerating effect.

**Figure 1:**
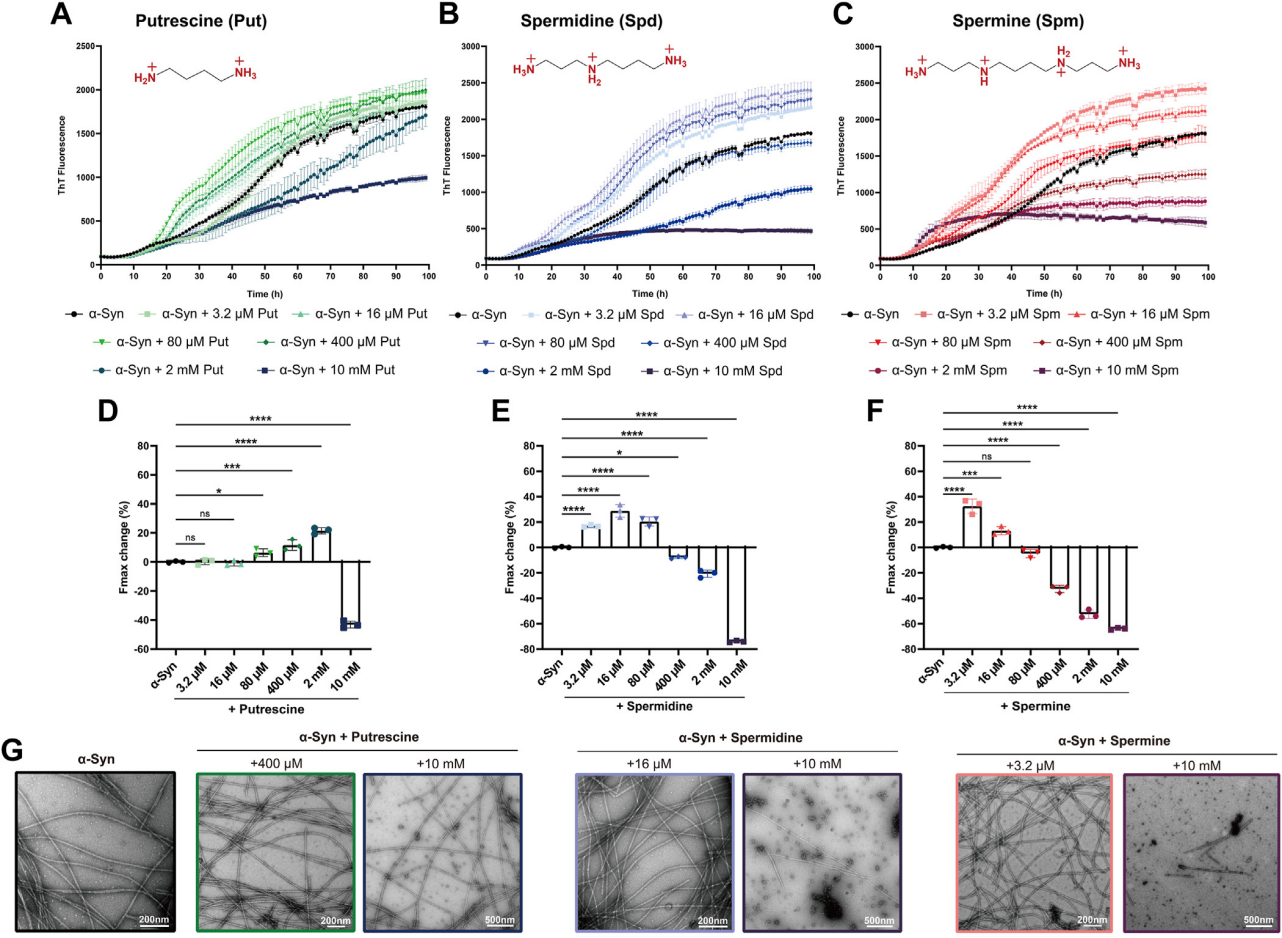
**Polyamines modulated the fibrillation kinetics of α-syn.***A–C*, ThT kinetic assay for α-syn aggregation in the presence of a gradient concentration of polyamines, (*A*) putrescine (Put), (*B*) spermidine (Spd), and (*C*) spermine (Spm). α-Syn concentration is 50 μM. Data are presented as mean ± standard deviation (SD). Results are from 3 independent experiments. *D–F*, the statistics analysis of max fluorescence (Fmax) for kinetic curves after 99 h incubation under each condition: (*D*) putrescine, (*E*) spermidine, and (*F*) spermine. (∗*p* < 0.05; ∗∗*p* < 0.01; ∗∗∗*p* < 0.001; ∗∗∗∗*p* < 0.0001; n.s., not significant, n = 3 biologically independent reactions, one-way ANOVA followed by Tukey’s *post hoc* test). *G*, representative NS-TEM images of samples taken from the endpoint of seven different conditions.

To validate our consumption, we then performed the polyamine ThT assay in the buffer without salt addition. Differently, we observed that low polyamine concentrations (putrescine: 80–400 μM; spermidine: 3.2–80 μM; spermine: 3.2–16 μM) significantly enhanced α-syn fibril formation. However, higher polyamine concentrations led to a decrement in fibrillation by reduced maximum fluorescence intensity (Fmax) (Fig. 1, *A*–*F*, Table S1.).

Interestingly, we found that the ability of polyamines to speed up α-syn fibrillation is positively related to their length and charge numbers. Spermine, with the longest length and maximum positive charge numbers among the tested polyamines, was highly effective in promoting α-syn fibrillation at relatively low concentrations. Using NS-TEM, we found that α-syn fibrils exhibited very similar morphologies, irrespective of the presence of polyamines. Furthermore, at increased polyamine concentrations of 10 mM, we observed a combination of spherical oligomers and short fibrils alongside the longer fibrils (Fig. 1G). Overall, our findings indicate that the impact of polyamines on α-syn aggregation is influenced by their concentration and charge.

Seeding experiments were performed to further discern the seeding properties of WT and spermine-induced α-syn fibrils. First, we sonicated the fibrils to obtain prepared preformed fibrils (PFFs) and characterized them *via* NS-TEM (Fig. S3*A*). Then ThT kinetics assay was employed to monitor the cross-seeding ability of α-syn^spermine^ PFFs. Seeding assays were conducted with varying seed concentrations (0.5%, 1%, or 2% w/w). Our findings indicate that α-syn^spermine^ seeds exhibit seeding ability comparable to that of α-syn seeds (Tris), albeit with a slightly extended lag time (Fig. S3*A*). This result suggests that α-syn seeds (Tris) and α-syn^spermine^ seeds shared similar structures and cross-seeding potency.

**Figure 2:**
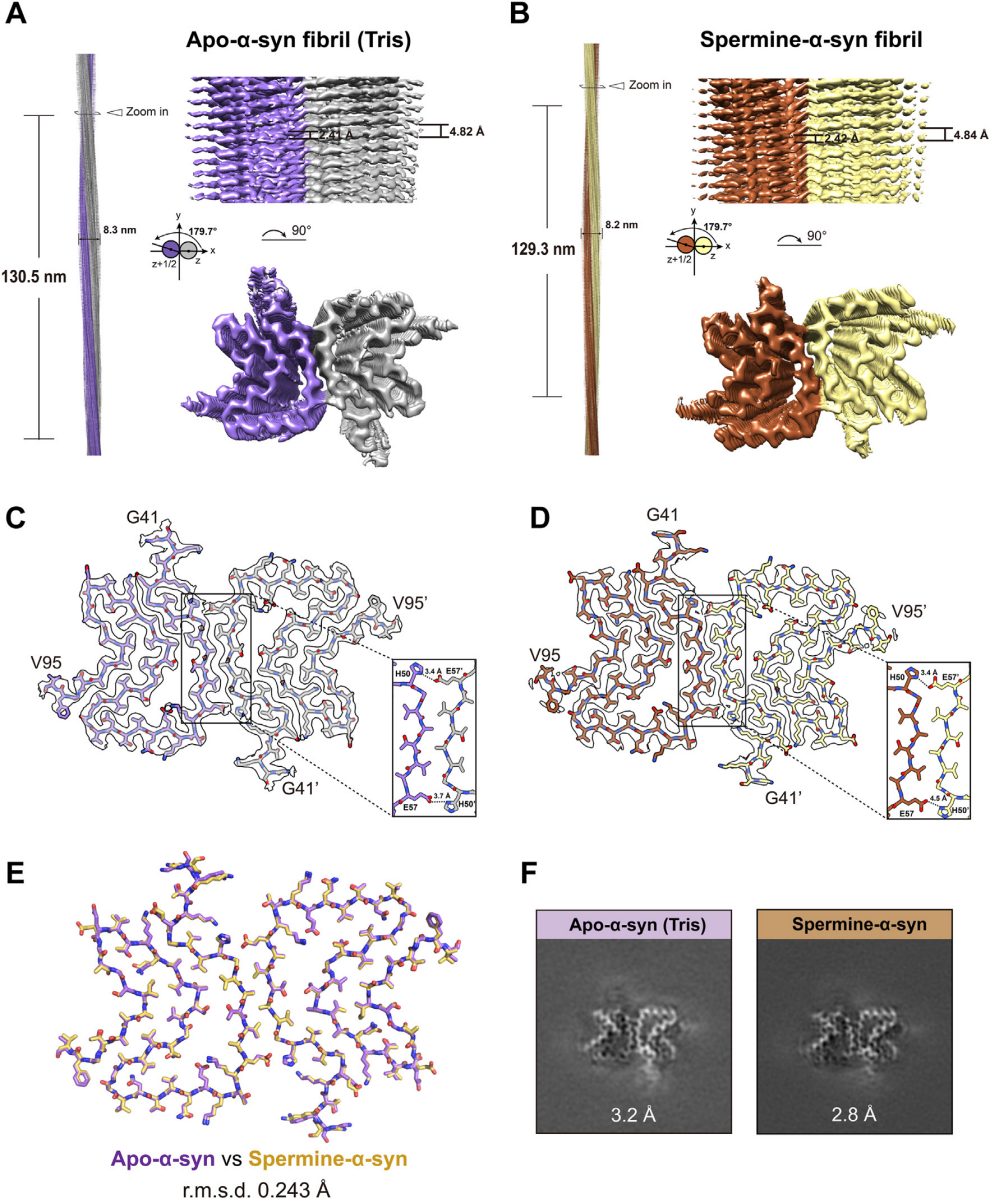
**Comparison of the cryo-EM structures of the apo-α-syn and spermine-α-syn fibrils.***A* and *B*, The reconstructed cryo-EM density maps of (*A*) apo-α-syn fibril, colored in *purple* and *grey*, and (*B*) spermine-α-syn fibril, colored in *brown* and *wheat*. Two protofilaments are colored separately. The fibril parameters including fibril widths, length of half pitches (180° helical turn), helical rises, and twist angles are indicated. The twist angles are graphically illustrated. *C* and *D*, cross-section views and interface regions of structural models of (*C*) apo-α-syn and (*D*) spermine-α-syn fitted in the density maps, respectively. The density maps are restricted to areas within a 2-Å radius of the structural models. *E*, structural comparison between apo-α-syn (colored in *purple*) and spermine-α-syn (colored in *yellow*). The structural models are shown in sticks with the main chain and side chain depicted. The r.m.s.d. between two structures is 0.243 Å over 101 Cα atoms (global alignment). *F*, central slices of 3D maps of apo-α-syn and spermine-α-syn fibril.

To further validate our hypothesis and explore the possible mechanism under this phenomenon, we sought to determine the atomic structure of polyamine-induced fibril. We prepared cryo-EM samples of α-syn fibrils formed with 3.2 μM spermine (termed spermine-α-syn fibril) and without spermine (termed apo-α-syn fibril). The cryo-EM micrographs were collected on a 300 kV Titan Krios microscope. For spermine-α-syn fibril, we analyzed 7608 fibrils picked from 1000 micrographs, while 5135 fibrils picked from 600 micrographs were examined for the apo-α-syn fibrils (Table. 1). Reference-free two-dimensional (2D) class averages revealed that spermine-α-syn fibril featured similar helical parameters as those of apo-α-syn fibril (Figs. 2, *A*, *B* and S4). The cryo-EM density maps (apo-α-syn fibril: 3.2 Å; spermine-α-syn fibril: 2.8 Å) (Fig. S5) and the structural models showed nearly identical conformation between the two types of fibrils, with a steric-zipper configuration maintained at the interface of the dimer (_50_HGVATVAE_57_) (Fig. 2, *C* and *D*). The steric-zipper interface is primarily stabilized by the hydrophobic interactions, with electrostatic interactions between E57 and H50 at the edge of the steric zipper core further stabilizing the interface. The all-atom root mean square deviation (r.m.s.d.) is 0.243 Å across 101 Cα atoms (Fig. 2*E*). No extra density was observed in 3D reconstructed map of the spermine-α-syn fibril, indicating spermine doesn't directly interact with the fibril core of α-syn (Fig. 2*F*). Therefore, spermine enhances fibrillation without changing the overall conformation of α-syn fibrils.

**Table 1:** Statistics of cryo-EM data collection, refinement and validation

Fibril type	Spermine-α-syn	Apo-α-syn (Tris)	PolyU-α-syn	PolyP-α-syn
PDB ID	8Y2Q	8Y2P	-	-
EMDB ID	EMD-38863	EMD-38862	-	-
Data collection				
Camera	BioContinuum K3	BioContinuum K3	BioContinuum K3	BioContinuum K3
Microscope	Krios G4	Krios G4	Krios G4	Krios G4
Magnification	105,000	105,000	105,000	105,000
Voltage (kV)	300	300	300	300
Defocus range (μm)	−1.0 to −2.0	−1.0 to −2.0	−1.0 to −2.0	−1.0 to −2.0
Pixel size (Å)	0.83	0.83	0.83	0.83
Exposure time (s/frame)	0.05	0.05	0.05	0.05
Number of frames	40	40	40	40
Total dose (e^-^/Å^2^)	55	55	55	55
Reconstruction				
Micrographs	1000	600	1000	1000
Picked fibrils	7608	5135	4154	1244
Box size (pixel)	360	360	-	-
Inter-box distance (Å)	26.9	26.9	-	-
Segments extracted	435,565	236,149	375,988	135,013
Segments after Class2D	196,614	132,626	-	-
Segments after Class3D	22,991	14,187	-	-
Resolution (Å)	2.8	3.2	-	-
Map sharpening B-factor (Å^2^)	−56.4402	−69.396	-	-
Pitch (nm)	129.34	130.51	-	-
Helical rise (Å)	2.415	2.408	-	-
Helical twist (°)	179.666	179.669	-	-
Atomic model				
Non-hydrogen atoms	2214	2214	-	-
Protein residues	330	330	-	-
Ligands	0	0	-	-
r.m.s.d. Bond lengths (Å)	0.006	0.011	-	-
r.m.s.d. Bond angles (°)	0.615	0.838	-	-
All-atom clash score	6.65	7.54	-	-
MolProbity score	1.75	1.67	-	-
Rotamer outliers	0.00%	0.00%	-	-
Ramachandran outliers	0.00%	0.00%	-	-
Ramachandran allowed	5.66%	3.77%	-	-
Ramachandran favored	94.34%	96.23%	-	-
Correlation coefficients (mask)	0.84	0.87

### PolyU induced various structural polymorphs of α-syn fibrils

**Figure 3:**
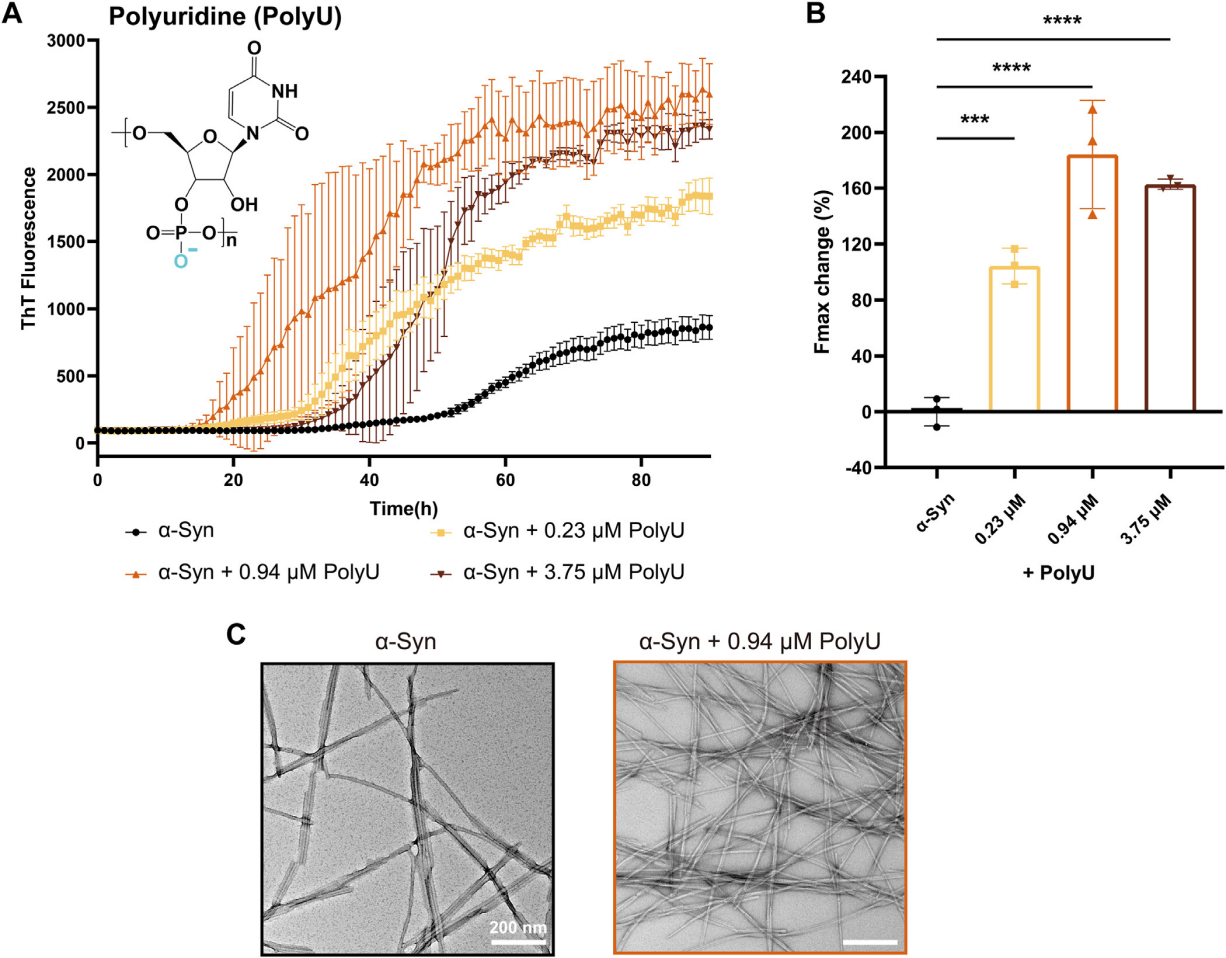
**PolyU promoted α-syn fibrillation.***A*, kinetics of α-syn fibril formation in the absence or presence of polyU monitored by a real-time ThT fluorescence assay. α-Syn monomer concentration is 50 μM. Results are the average of three independent experiments, and the error bars represent SD. *B*, Percentage change of Fmax for kinetic curves after 90 h detection in each condition. (∗∗∗*p* < 0.001; ∗∗∗∗*p* < 0.0001, one-way ANOVA followed by Tukey’s *post hoc* test). *C*, representative NS-TEM images at the endpoint of the control group (*black*) and 0.94 μM polyU group (*brown*).

We further investigated the effects of polyanions on α-syn fibrillation. Considering the known interaction between α-syn and RNA, as well as RNA's role in modulating phase separation and fibrillation in various amyloid proteins, we chose polyU as a representative RNA-like polyanion for investigation (21, 32, 33, 34, 35). We evaluated the impact of polyU on α-syn fibrillation *via* the ThT kinetic assay in a buffer of 50 mM Tris-HCl at pH 7.5, with 150 mM KCl. Our findings showed that polyU significantly boosted α-syn fibrillation in a dose-dependent manner. By examining the growth curve and changes in Fmax at different polyU concentrations, we found that Fmax peaked at 0.94 μM and decreased at higher concentrations (3.75 μM) (Fig. 3, *A* and *B*). This acceleration effect of polyU on α-syn fibril formation was then visualized by NS-TEM imaging and western blots assays (Figs. 3C, S6, *A* and *B*).

**Figure 4:**
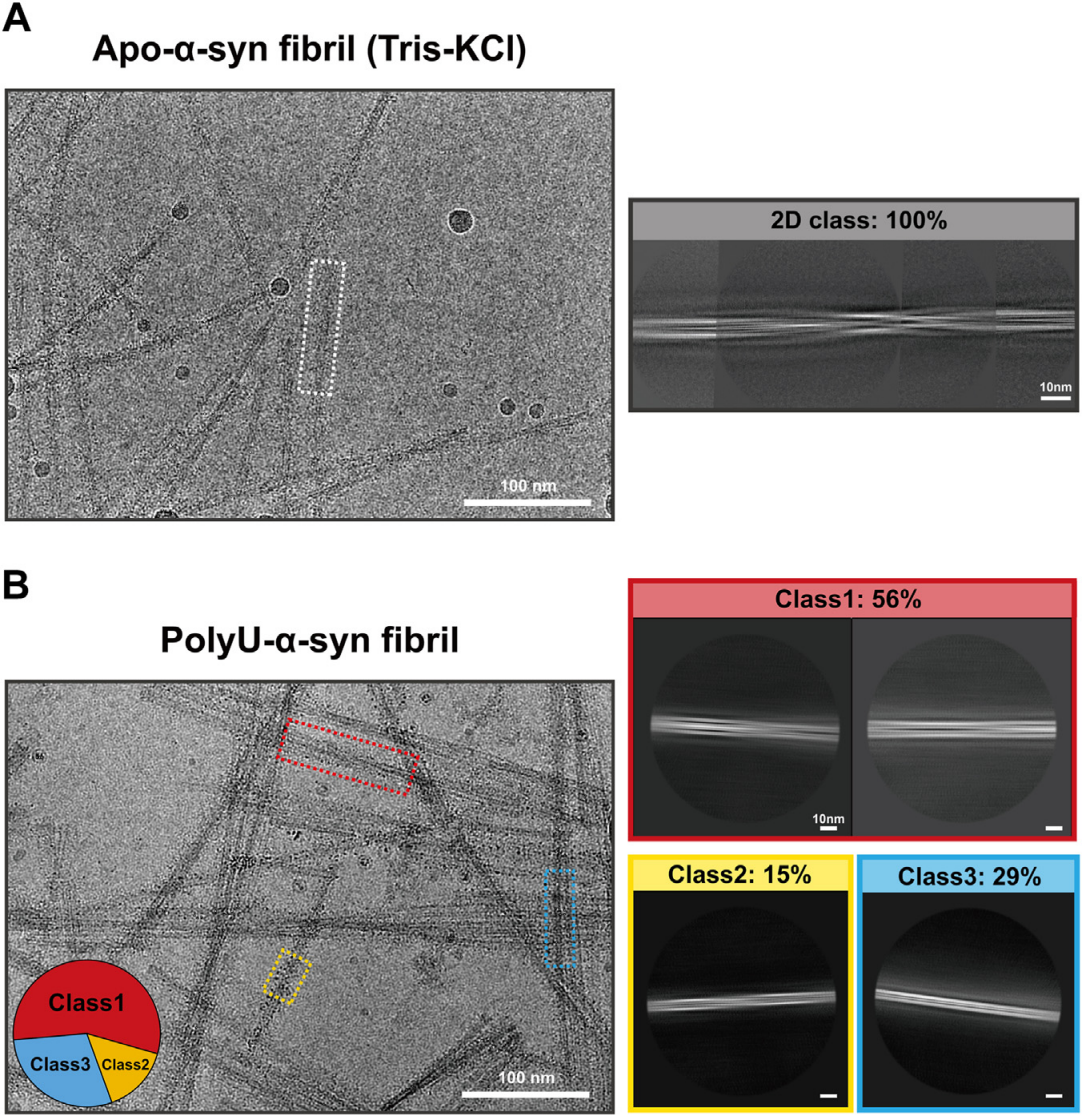
**PolyU induced α-syn to form different morphologies of fibrils.***A*, representative cryo-EM micrograph of the apo-α-syn fibrils (Tris-KCl). 2D class averages comprising a half helical crossover of apo-α-syn fibril is enclosed in a *white* dotted box and zoom in on the *right*. *B*, cryo-EM micrograph of the polyU-α-syn fibrils. Different classes are enclosed in dotted boxes colored corresponding to representative 2D classes on the *right*. Circular pie chart shown the distribution of different 2D classes. Class1 (*red*) and class2 (*yellow*) are two types of twisted fibrils. Class3 (*blue*) is untwisted.

In contrast to the uniform fibrils generated by polyamines (Figs. 1*G* and 2), the α-syn fibrils induced by polyU (termed polyU-α-syn fibril) displayed a wide range of morphologies, including both slender and bundled fibrils (Fig. 4). We then analyzed the cryo-EM structure of polyU-α-syn fibril. We manually selected 4154 fibrils from 1000 cryo-EM images. Interestingly, the 2D classification of polyU-α-syn fibril identified three distinct morphological types: ∼71% of the fibrils (class1 and class2) were twisted, with the 15% in class 2 displaying a slower twisting morphology; and the remaining 29% showed no apparent twist (Fig. 4*B*). Each type of fibril polymorph exhibited significant differences from the unitary twisted morphology of the apo-α-syn fibril (Fig. 4). Despite these findings, the 3D structure of polyU-α-syn fibril remained elusive, as the absence of a regular crossover distance in the 2D class averages precluded its determination. Nonetheless, based on ThT kinetics and the observed 2D class averages, our results convincingly demonstrate that polyU not only accelerates α-syn fibrillation but also induces the formation of polymorphic fibril structures.

### PolyP induced formation of untwisted α-syn fibrils

**Figure 5:**
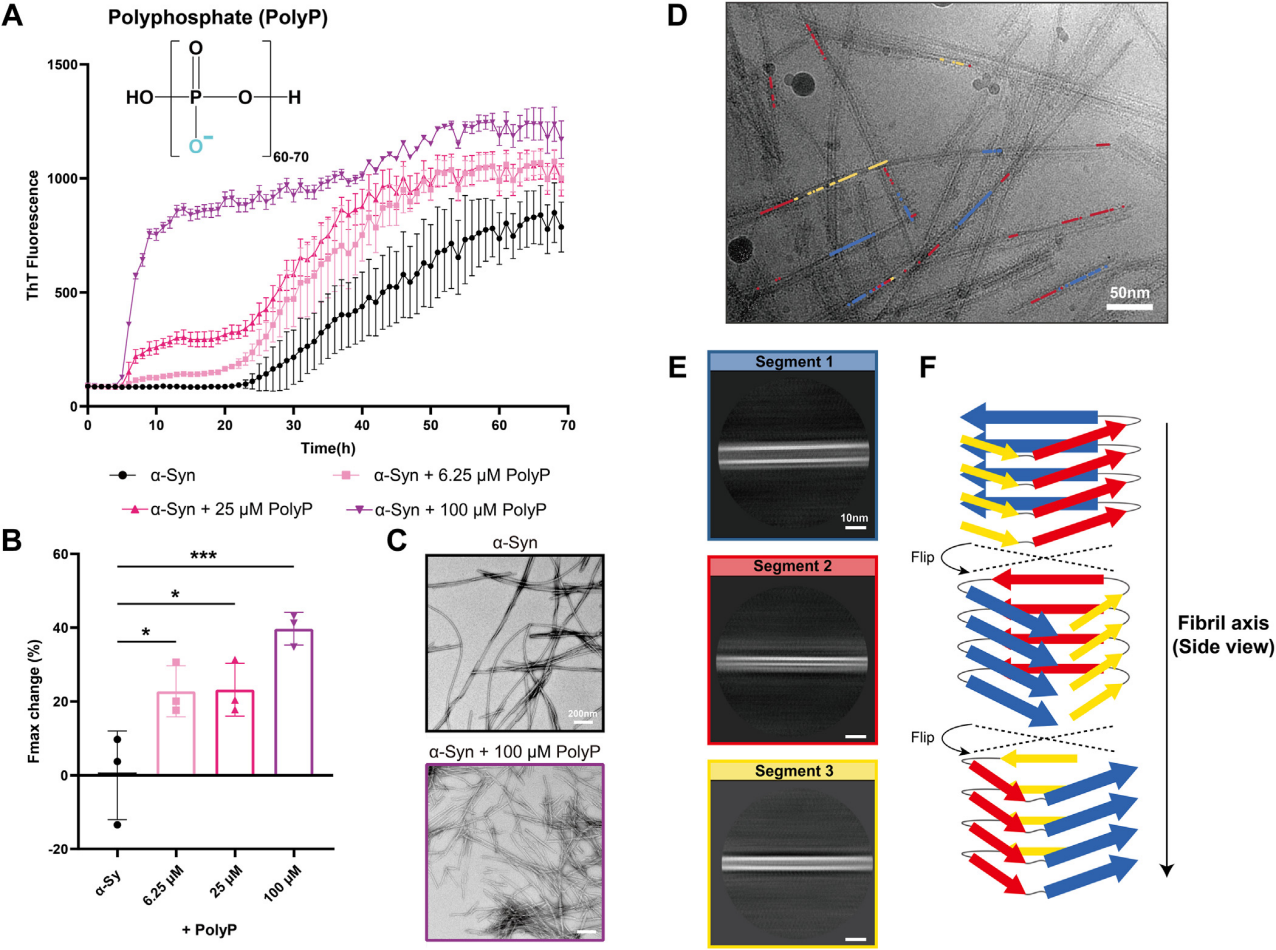
**Untwisted α-syn fibril formed in the presence of polyP.***A*, ThT fluorescence measurements of 50 μM α-syn incubated with or without polyP. Results are the average of three independent experiments, and the error bars represent SD. *B*, Fmax changes for kinetic curves in each condition after 70 h detection. ∗*p* < 0.05; ∗∗∗*p* < 0.001, one-way ANOVA followed by Tukey’s *post hoc* test. *C*, Representative NS-TEM images of α-syn fibrils formed in the absence or presence of polyP. *D* and *E*, Cryo-EM micrographs of α-syn fibrils induced by 100 μM polyP (*D*). Representative 2D class averages of fibrils with different diameters (*E*) are colored corresponding to fibril segments in panel D. *F*, a proposed model of an untwisted polyP-α-syn fibril (side view), illustrating possible flips upon fibril elongation which may result in a heterogeneous structural arrangement as observed in panel (*D* and *E*).

Following the significant influence of polyU on α-syn fibrillation kinetics and fibril structures, we explored whether other polyanions could exert similar or divergent effects. PolyP, known for its high negative charge density, has been previously implicated in affecting α-syn fibrillation (29, 30, 31). We investigated the potentiality of polyP to alter the α-syn structural polymorphs during fibrillation. α-Syn fibrillation assay was carried out by titrating polyP (containing 60–70 phosphates) concentrations in 50 mM Tris-HCl buffer at pH 7.5, with 150 mM KCl. Aligning with earlier findings (29, 30, 31), polyP markedly accelerated α-syn fibrillation in a concentration-dependent manner, evident from the shortened lag phase and increased Fmax value (Fig. 5, *A* and *B*). Western blots were also applied to confirm the increase in the amount of α-syn fibrils in the presence of polyP (Fig. S6, *C* and *D*). Crucially, TEM imaging revealed that polyP not only significantly increased the overall quantity of fibrils but also led to the emergence of fibrils with new morphologies, characterized by a slightly thicker and shorter structure, as well as fibril bundling and partial aggregation (Fig. 5, *C* and *D*).

Similarly, we conducted the seeding assay to explore the cross-seeding capacity of polyanion-induced α-syn fibrils. Interestingly, while α-syn^PolyU^ PFFs significantly accelerate α-syn aggregation, α-syn^PolyP^ seeds did not speed up α-syn fibrillation (Fig. S3*B*). Considering that spermine does not alter the overall structure of α-syn and α-syn^spermine^ seeds show no difference in seeding capacity (Fig. S3*A*), our results further indicate that polyP leads to the formation of structurally distinct strains with a less effective seeding ability.

To elucidate the high-resolution structure, we utilized cryo-EM to analyze 2144 polyP-induced α-syn fibrils (termed polyP-α-syn fibril) from 1000 cryo-EM images. Strikingly, 2D classification showed that polyP-α-syn fibrils predominantly formed untwisted structures with varying diameters (Fig. 5*E*), in stark contrast to the uniform twisted structure of α-syn fibrils formed without polyP (Fig. 4*A*). However, due to the limitation of the helical reconstruction procedure adopted here to resolve amyloid fibril structure, we were unable to reconstruct the untwisted fibril without obvious helical parameters (36, 37, 38, 39). Although lacking atomic structures of these untwisted fibrils we were able to categorize three distinct fibril segments based on morphological variations observed in the 2D class averages. Intriguingly, we observed that different 2D classes could coexist within a single fibril (Fig. 5, *D* and *E*). This phenomenon may be attributed to the 2D class averages representing different side views of one fibril, which undergoes flips during elongation (Fig. 5*F*). These results suggest a heterogeneous structural arrangement, potentially induced by polyP.

In conclusion, our findings suggest that diverse polyanions not only alter the α-syn fibrillation kinetics but also give rise to distinct α-syn fibril polymorphs. This implies that various polyanions may influence α-syn fibrillation through unique mechanisms.

## Discussion

α-Syn is capable of forming various structural polymorphs with distinct pathogenic profiles, both under *in vitro* conditions and in the brains of patients with different synucleinopathies (37, 38, 40, 41, 42, 43). This phenomenon is believed to contribute to the varied clinical and pathological manifestations seen in disorders such as PD, Multiple System Atrophy (MSA), and other related disorders (44, 45, 46, 47). Notably, unidentified non-proteinous densities were frequently observed in the structures of α-syn fibrils extracted from the brains of PD and MSA patients (37, 38, 42). These extra densities aligned along the fibril axis, located either adjacent to the fibril surface or within its core, hinting at the involvement of cofactors in the formation of these distinct fibril structures. Considering the naturally charged properties of α-syn, variously charged biopolymers are potential candidates that interact with α-syn within a cellular context and may play a role in α-syn fibrillation. Specifically, polyamines are positively charged molecules abundantly found in synaptic vesicles, which can be released to the synaptic cleft to regulate ion channels (24). Moreover, the presence of mitochondrial membrane fragments in PD brains with Lewy pathology suggests a potential interaction between α-syn and mitochondrial contents such as RNA (15). Additionally, inorganic polyphosphate, known to exist both intra- and extracellularly, is found to be secreted by astrocytes and taken up by neurons, indicating its widespread presence and potential role in α-syn aggregation (48).

In this study, we explored the structural mechanisms by which different biopolymers, including polycations and polyanions, affect the kinetics and structures of α-syn fibrils. Intriguingly, we discovered that while various biopolymers commonly expedite α-syn aggregation at low stoichiometry, they would play distinct roles in the α-syn fibrillation process. Specifically, spermine does not change the structure of α-syn fibrils, whereas polyU and polyP lead to the formation of different α-syn fibril polymorphs.

The highly negatively charged C-terminal region of α-syn is known to engage in transient interactions with the NAC and N-terminal regions, which plays a role in inhibiting α-syn fibrillation (14, 49). It has been reported that polyamines mainly interact with the C-terminal region of α-syn through electrostatic interaction (50). This interaction could disrupt the C-terminal's binding with the N-terminal and the NAC regions, rendering α-syn more susceptible to aggregation. Additionally, since the C-terminal region does not contribute to fibril core formation (51, 52), polyamines may influence aggregation kinetics without altering the overall fibril structure. Conversely, polyanions are known to directly interact with positively charged “KTK” motifs residing at the N-terminal and NAC regions that are critical for α-syn fibril core formation (31). Thus, polyanions might affect both the kinetics and structures of the fibrils. Due to the varied structures and properties of polyanions, they may bind to positively charged residues located at different sites of α-syn, further inducing the formation of distinct structural polymorphs. Although we have observed distinct twisted 2D class averages in polyU-α-syn fibril, our inability to resolve the 3D structure of the polyU-α-syn fibril might be attributed to the inherent heterogeneity of polyU, which exhibits varying degrees of polymerization. This heterogeneity likely affects the uniformity of the α-syn fibrils, complicating the determination of the 3D structure. Besides, the lack of twist precluded the determination of the three-dimensional structure of polyP-α-syn-fibril by cryo-EM. To fully grasp how different polyanions generate diverse fibril structures, it is essential to conduct further studies aimed at elucidating atomic structures of α-syn fibril polymorphs induced by polyanions. Overall, by determining the structures of biopolymer-induced α-syn fibrils, our work revealed the intricate and critical roles of different charged biopolymers in the process of α-syn fibril formation.

## Experimental procedures

### Preparation of WT α-syn monomer

Recombinant N-terminally acetylated human α-syn wild type (WT) was over-expressed and purified as previously established protocol (43). In brief, the plasmid was transformed into *E.coli* BL21 (DE3) and α-syn expression was induced by 1 mM isopropyl-1-thio-D-galactopyranoside (IPTG) at 37 °C for 4 h. Post-induction, bacteria were harvested at 4000 rpm for 20 min, and the pellet was lysed in 100 mM Tris-HCl at pH 8.0, with 1 mM EDTA, and 1 mM phenylmethylsulfonyl fluoride (PMSF). The supernatant was then boiled for 10 min and subjected to another round of centrifugation. To this supernatant, streptomycin (20 mg/ml) was added to precipitate nucleic acids, followed by another centrifugation. Subsequently, the pH of the supernatant was adjusted to 3.5 with 2 M HCl to precipitate other proteins. Then the supernatant was dialyzed overnight in 25 mM Tris-HCl at pH 8.0 at 4 °C. Final purification was achieved using Q column (GE Healthcare, 17–5156–01), followed by Superdex 75 column (GE Healthcare, 28–9893–33).

### Commercial chemical compounds

Polyamines, including putrescine (CAS No. 110–60–1), spermidine (CAS No. 124–20–9), and spermine (CAS No. 71–44–3), were purchased from MedChemExpress (MCE). PolyU (CAS No. 27416–86–0) was purchased from Sigma-Aldrich and polyP was a gift from Dr Jing Zhao. Polyamines were dissolved into deionized distilled water (ddH_2_O) to obtain a 500 mM stock solution. PolyU and polyP were dissolved into ddH_2_O with a stock concentration of 10 mg/ml.

### ThT kinetic assay

To monitor the α-syn aggregation kinetics in the presence of varying biopolymers, ThT fluorescence assay was conducted. For polycation-induced α-syn reactions, 50 μM α-syn monomer, 50 μM ThT, and a series of concentrations of polyamines were mixed in a fibrillation buffer (50 mM Tris-HCl, pH 7.5, with or without 150 mM KCl). In the case of polyanion-induced α-syn reactions, different concentrations of polyU and polyP were added to α-syn monomer (50 μM) with 50 μM ThT under the condition of 50 mM Tris-HCl at pH 7.5, with 150 mM KCl. These reactions were performed in a black 384-well plate with a clear bottom (ThermoFisher Scientific, 142761). The plate was incubated with continuous shaking (900 rpm, orbital) at 37 °C in the BMG FLUOstar Omega plate reader. Fluorescent intensities were recorded hourly at an excitation wavelength of 440 nm and an emission wavelength of 480 nm. Kinetic curves were plotted using GraphPad Prism 9, and maximum intensities were determined through nonlinear regression, as previously described (53).

### Negative-staining transmission electron microscopy (TEM)

For TEM analysis, a 3 μl aliquot of each fibril solution sample was loaded on a glow-discharged 230 mesh grid with carbon support film (Beijing Zhongjingkeyi Technology Co., Ltd) for 45 s. The excess sample was removed using filter paper, followed by a wash with 5 μl ddH_2_O. The grid was then stained with 5 μl uranyl acetate (2%, v/v) for 45 s and air-dried before imaging. TEM images were acquired using Tecnai G2 spirit transmission electron microscope with 120 kV voltage (FEI Company) equipped with a 4000 × 4000 charged-coupled device camera (BM-Eagle, FEI Tecnai).

### Western blotting

ThT reaction samples were collected and centrifugated at 14,000 rpm for 1 h. The supernatant and pellet were then used as samples for immunoblotting. For the supernatant, buffer containing 50 mM Tris-HCl at pH 7.5, with 150 mM KCl (termed as Tris-KCl buffer) was added to adjust to the same volume. For the pellet, the samples were resuspended in an equal volume of Tris-KCl buffer and sonicated for 60 cycles (1 s on/off per cycle) under 20% amplitude (JY92-IIN sonicator). The sonicated fibril samples were then dissolved in 2M urea, 2% sodium dodecyl sulfate, and boiled for 90 min. Then all samples were boiled at 100 °C for 10 min with the SDS-PAGE loading dye and loaded on 4 to 20% Bis-Tris gels (GenScript), followed by electrophoresis at 140 V for 45 min. Proteins were then transferred to a PVDF membrane (Millipore). After being blocked with Protein Free Rapid Blocking Buffer (Epizyme) in 1X Tris-buffered saline, with 0.5% (v/v) Tween20 (TBST) for 15 min at room temperature, membranes were incubated with Anti-Alpha-synuclein antibody (Abcam, ab138501) dilute 1:2000 in 5% BSA in TBST overnight, then washed three times in TBST. Then, membranes were incubated for 1 h with Goat anti-Rabbit IgG HRP (Invitrogen, Catalog No.31460) diluted 1:10,000 in 5% milk in TBST. Membranes were washed three times in TBST. Super ECL Detection Reagent (Yeasen) was applied to membranes. Membranes were imaged using an iBright 1500 (Invitrogen).

### Seeding assay

Polymer-induced α-syn fibrils were formed using 50 μM α-syn monomer in the absence and presence of different biopolymers: 3.2 μM spermine, 0.94 μM polyU, and 100 μM polyP. α-Syn PFFs seeds were prepared by sonication at 20% power for 40 times (1 s on/off per cycle) on ice by JY92-IIN sonicator. The concentration of sonicated PFF seeds was calculated as equal to that of the PFFs before sonication. The concentration of PFFs before sonication was calculated as the amount of α-syn monomer subtracting the amount of soluble α-syn after fibril formation. Subsequently, WT or α-syn^biopolymer^ PFFs at concentrations of 0.5%, 1% or 2% w/w α-syn were added to 50 μM α-syn monomer with 50 μM ThT to monitor the aggregation kinetics. Seeding assay of fibrils formed in the presence of α-syn^spermine^ PFFs was carried out in 50 mM Tris-HCl, pH 7.5. Seeding assay of polyanion-induced fibrils was performed in Tris-KCl buffer.

### Preparation of α-syn fibrils for cryo-EM

For polyamine-induced cryo-EM sample, α-syn monomer (50 μM, in buffer containing 50 mM Tris-HCl, pH 7.5, 0.05% NaN_3_) was incubated at 37 °C with constant agitation at 900 rpm in ThermoMixer (Eppendorf) in the absence or presence of 3.2 μM spermine. In parallel, 50 μM α-syn monomer (in buffer containing 50 mM Tris-HCl, pH 7.5, 150 mM KCl, 0.05% NaN_3_) was incubated with either 0.94 μM polyU or 100 μM polyP at 37 °C with constant agitation (900 rpm). The mature fibrils were subsequently collected for cryo-EM analysis.

### Cryo-EM data collection

The prepared α-syn fibrils were diluted to 3 μM by the fibrillation buffer. A 4 μl aliquot of the fibrils-containing solution was applied to a glow-discharged holey carbon Cu electron microscope grid (Quantifoil R1.2/1.3, 300 mesh) twice. Then, the cryo-EM grids were plunge-frozen in liquid ethane using Vitrobot Mark IV (FEI, Thermo).

For image acquisition, a BioContinuum K3 direct detector (Gatan, Inc.) in counting mode on a Thermo Fisher Titan Krios G4 cryo transmission electron microscope (Thermo Scientific) at 300 kV was used, using a GIF Quantum energy filter (Gatan, Inc.) with a slit width of 20 eV to remove inelastically scattered electrons. 40 frame movies per micrograph were recorded at × 105,000 magnification with a pixel size of 0.83 Å pixel^-1^ and the total dose was ∼55 e−/Å^2^ with an exposure time of 2.0 s. Automated cryo-EM data collection was performed by using EPU software (Thermo Scientific) with defocus values from −1.0 to −2.0 μm.

### Cryo-EM image pre-processing and helical reconstruction

For image pre-processing, 40 movie frames per micrograph were corrected for beam-induced motion, aligned, dose-weighted, and further binned with a physical pixel size of 0.83 Å using MotionCorr2 (54). The contrast transfer function was estimated from motion-corrected images by CTFFIND-4.1.8 (55). Helical reconstruction was performed in RELION (56, 57).

### Apo-α-syn (Tris) datasets

Helical reconstruction of apo-α-syn (Tris) was performed in RELION 3.1 (56). 5135 fibrils were manually picked from 600 micrographs then extracted to segments with a box size of 288 pixels and an inter-box distance of 23.9 Å. The particles were then re-extracted with 864 box size and downscaled to 360 pixels. Then, reference-free 2D classification steps with a decreasing in-plane angular sampling rate from 4 to 0.5 and a T = 2 regularization parameter were performed to calculate the apparent half pitches from the 2D class averages and discard the segments contributing suboptimal 2D class averages. An initial 3D model was constructed *de novo* from 2D class averages of the purified segments that comprise the entire helical crossover using the relion_helix_inimodel2d program (56). The purified segments were re-extracted using a box size of 360 pixels. These segments and the initial 3D model that was low-pass-filtered to 30 Å were further applied to perform 3D classifications (k = 1) using the helical parameters calculated through the splicing of 2D class averages. The best 3D reconstruction map was selected and subjected to additional rounds of 3D classifications (k = 3 followed by k = 1, and final round of k = 3) with local optimization of helical twist and rise while β-strands perpendicular to the helical axis were clearly separated. Then 3D auto-refinement with optimization of helical twist and rise after reconstructions was carried out.

Finally, the map was sharpened with a soft-edge solvent mask using the standard “post-processing” program in RELION 3.1 (56). Overall resolution estimate was calculated based on the gold-standard 0.143 Fourier shell correlation (FSC) between the two independently refined half-maps. Then the local resolution was estimated using the “Local resolution” procedure in RELION 3.1 with the same mask and B-factor in post-processing (56).

### Spermine-α-syn datasets

7608 fibrils were manually picked from 1000 micrographs and extracted using an inter-box distance of 23.9 Å and a box size of 288 pixels using RELION 3.1 (56). Then particles were re-extracted with 1050 pixels scaled down to a 448-pixel box size. 2D classification with a decreasing in-plane angular sampling rate from 8 to 0.5 and a T = 2 regularization parameter was performed with all particles to calculate the apparent half pitch. After discarding the segments contributing suboptimal 2D class averages, an initial 3D model was constructed using the relion_helix_inimodel2d program (56). The purified segments were re-extracted using a box size of 512 pixels for 3D classification with k = 3 followed by k = 1. The best 3D classes were used as the initial model for subsequent 3D classifications with smaller box-size particles (360 pixels). Two rounds of 3D classification with k = 3 and one round with k = 1 were performed. Consequently, 22,991 particles from the best 3D class were selected for high-resolution 3D refinement. Sharpened map overall resolution estimate was processed in the same way as apo-α-syn (Tris) datasets.

### PolyU-α-syn datasets

A total of 4154 fibrils were manually picked using RELION 4.0 from 1000 micrographs and were segmented into 375,988 particles with a box size of 288 Å (57). After re-extraction into 960 pixels scaled down to a 360-pixel box size, reference-free 2D classification was performed at a T = 2 regularization parameter with an in-plane angular sampling rate of 2°. Segments displaying clear structure features were selected and re-extract to 1200 pixels box size for further classification (in-plane angular sampling rate of 1°). Finally, the best 2D classes were further selected for processing using a box size of 1600 pixels and an in-plane angular sampling rate of 0.5°.

### PolyP-α-syn datasets

For analyzing polyP-α-syn datasets, 1244 manually picked fibrils from 1000 micrographs were first extracted to segments with a box size of 288 pixels. Subsequently, the segments were re-extracted with a box size of 960 pixels. Then, reference-free 2D classification was carried out with an in-plane angular sampling rate of 2 and a T = 2 regularization parameter.

### Atomic model building

The starting model of α-syn was based on the structure of α-syn polymorph 1a fibril (PDB ID: 6A6B) (43). The starting coordinates for 3 rungs of α-syn fibril core were manually docked into the central region of the sharpened density map in Chimera (58).

Then the three-layer protein model was manually adjusted in COOT (59), followed by refinement against the corresponding map by phenix.real_space_refine program in PHENIX with secondary structure and geometry restraints (60). Additional details for helical reconstruction and model building were shown in Table 1.

## Data availability

The cryo-EM map of spermine-α-syn and apo-α-syn fibril had been deposited in the Electron Microscopy Data Bank (EMDB) under accession numbers EMD-38863 and EMD-38863. The corresponding refined atomic models had been deposited in the Protein Data Bank (PDB) following accession numbers PDB-8Y2Q and PDB-8Y2P, respectively. The structural models used in this study are available in the PDB database under accession codes 6A6B.

## Supporting information

This article contains supporting information.

## Conflict of interests

The authors declare that they have no conflicts of interest with the contents of this article.
