# Supplementary material for: Different charged biopolymers induce α-synuclein to form fibrils with distinct structures
Source: J Biol Chem. 2024 Oct 5;300(11):107862. doi: 10.1016/j.jbc.2024.107862 (PMC11570948; doi:10.1016/j.jbc.2024.107862)
Supplement: Supplementary Figures S1–S6 [file mmc1.docx]

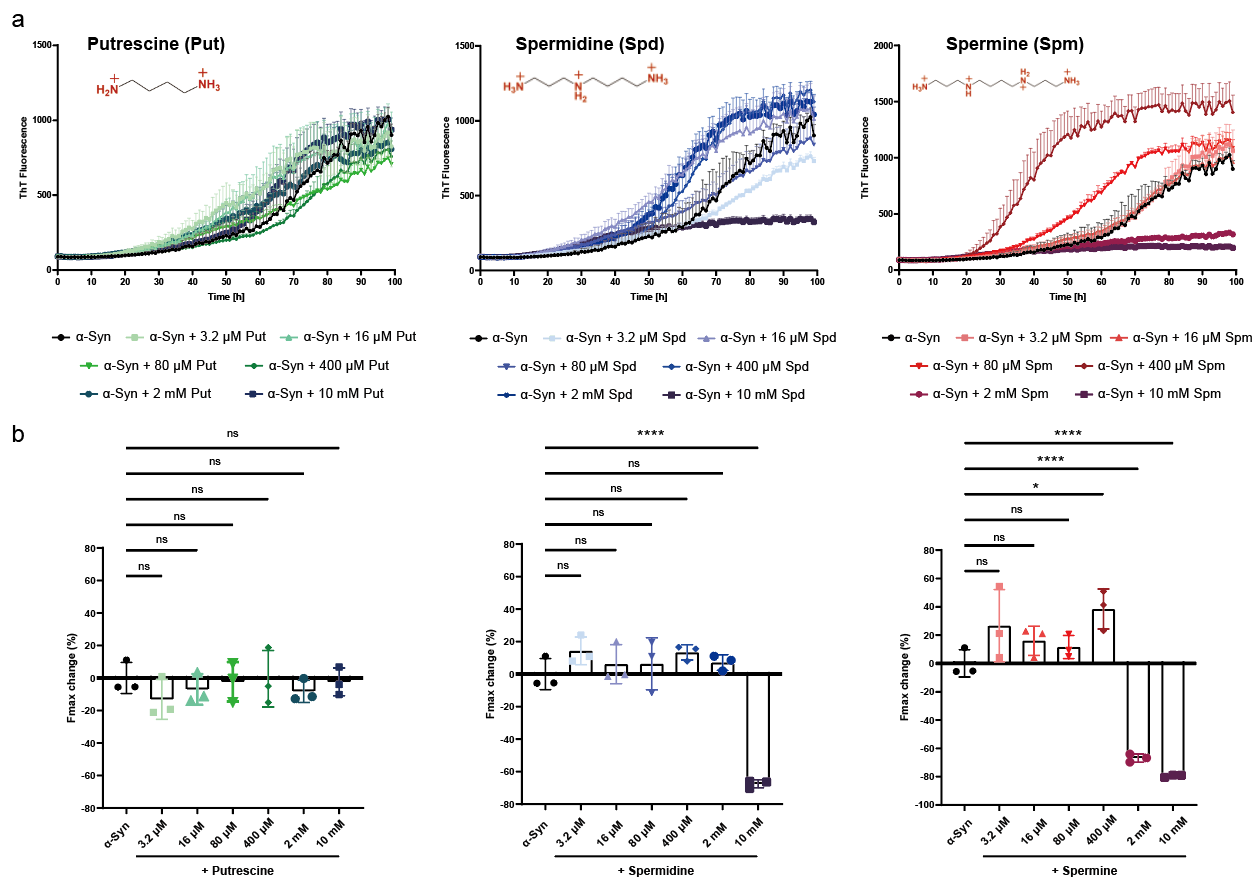


**Figure S1. ThT kinetic assay of polyamines-induced α-syn fibrillation in the absence of KCl.**

(**a-c**) ThT kinetic assay for α-syn aggregation in the presence of a gradient concentration of polyamines, (**a**) putrescine (Put), (**b**) spermidine (Spd), and (**c**) spermine (Spm). α-Syn concentration is 50 μM. Data are presented as mean + SD. Results are from 3 independent experiments. (**d-f**) The statistics analysis of max fluorescence (Fmax) for kinetic curves after 99 hours incubation under each condition: (**d**) putrescine, (**e**) spermidine, and (**f**) spermine. (*P < 0.05; **** P < 0.0001; n.s., not significant, n = 3 biologically independent reactions, one-way ANOVA followed by Tukey’s post-hoc test).

**
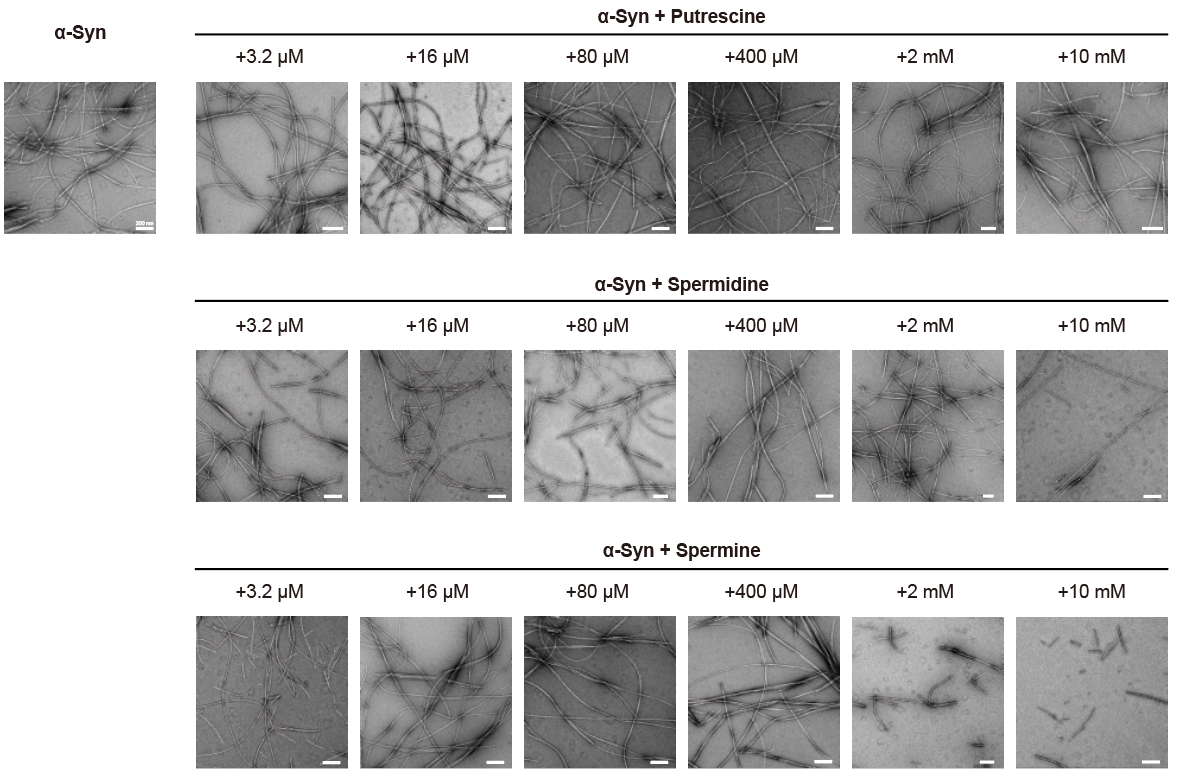
**

**Figure S2. Representative NS-TEM images of samples taken from the endpoint of each reaction of ThT assays in the absence of KCl.**

*
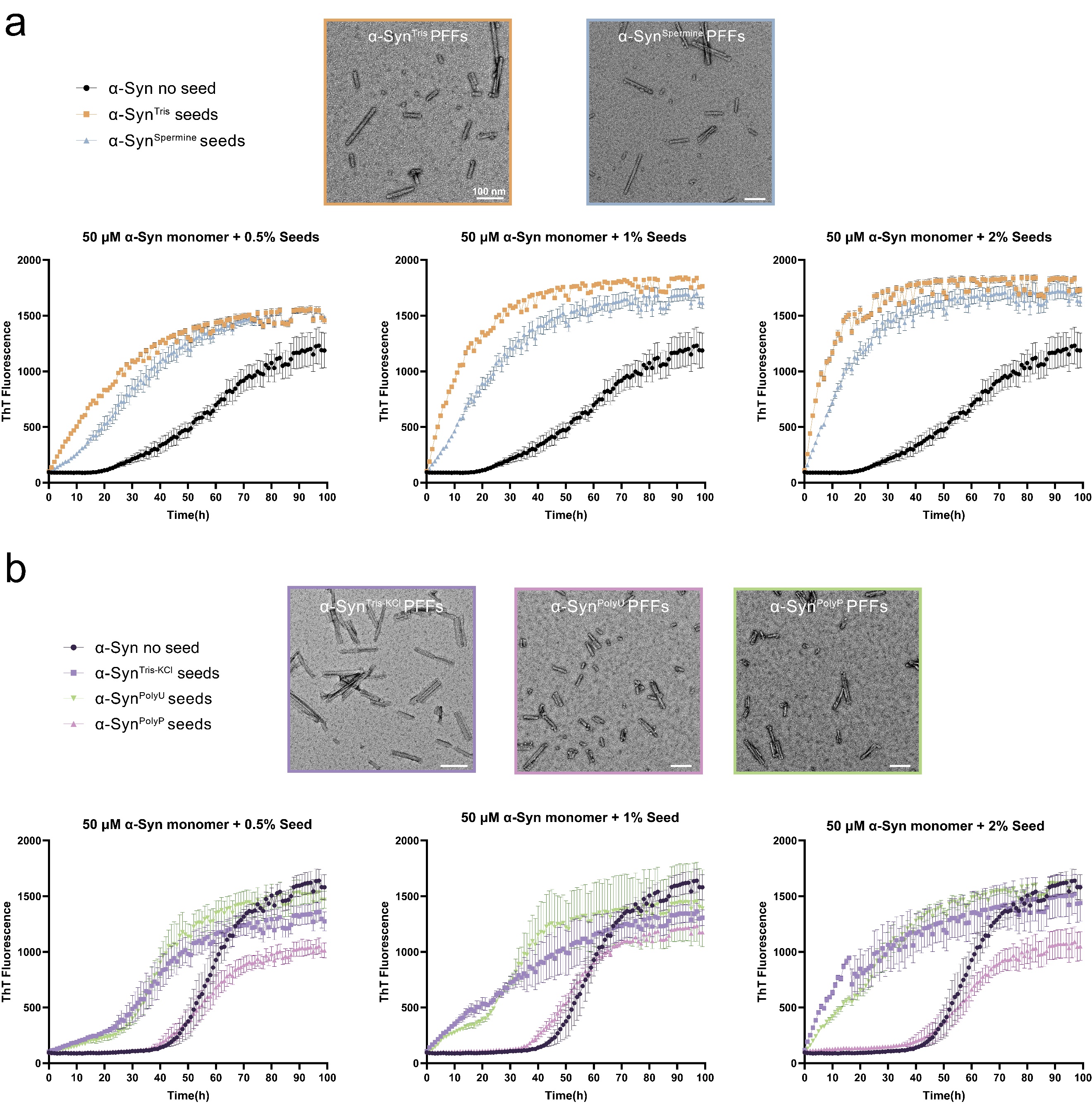
*

**Figure S3. Seeding ability of different α-syn^polymer^ PFFs.**

NS-TEM images of α-syn PFFs (Tris) and α-syn^polymer^ PFFs after sonication. ThT kinetic assay of the WT α-syn fibril formation with or without seeding. Mole percent of added PFF seeds are indicated. Data shown are mean ± SD, n = 3.


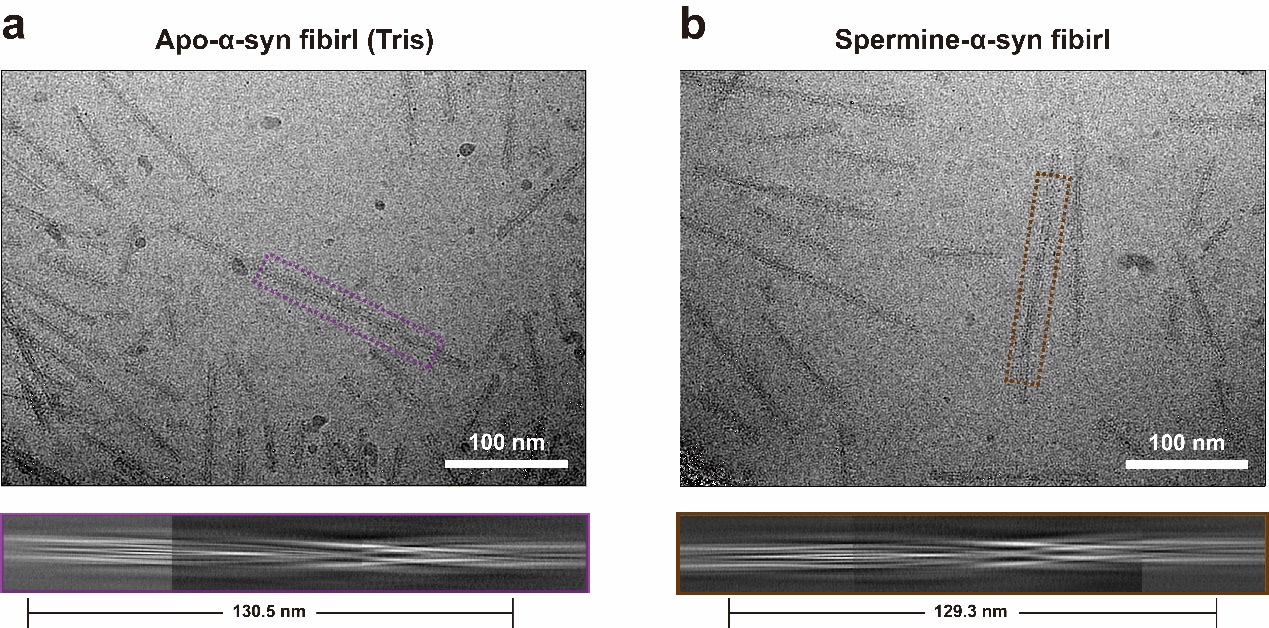


**Figure S4. Representative cryo-EM micrographs and half crossovers of apo-α-syn fibril (Tris) and spermine-α-syn fibril.**

(**a**) Cryo-EM micrograph of the apo-α-syn fibrils (Tris). 2D class averages with an 864-pixel box size comprising a half helical crossover is shown at the bottom. (**b**) Cryo-EM micrograph of the spermine-α-syn fibril, with 1050-pixel box size 2D class averages spanning a half helical crossover below.

**

**

**Figure S5. Resolution estimation of the cryo-EM structures of apo-α-syn fibril (Tris) and spermine-α-syn fibril.**

Left: Gold standard Fourier shell correction curves (FSC) of two independently refined half-maps of (**a**) apo-α-syn fibril (Tris) and (**b**) spermine-α-syn fibril are shown in black; FSC curves of the final refined atomic model against the final cryo-EM map are shown in red; FSC curves between a model refined against half map 1 are shown in dashed blue; FSC curves of the same model against half map 2 are shown in dashed orange. The overall resolution of apo-α-syn fibril and spermine-α-syn fibril is 3.2 Å and 2.8 Å, respectively. Right: Local resolution estimation for the recombinant density map of (**a**) apo-α-syn fibril (Tris) and (**b**) spermine-α-syn fibril.


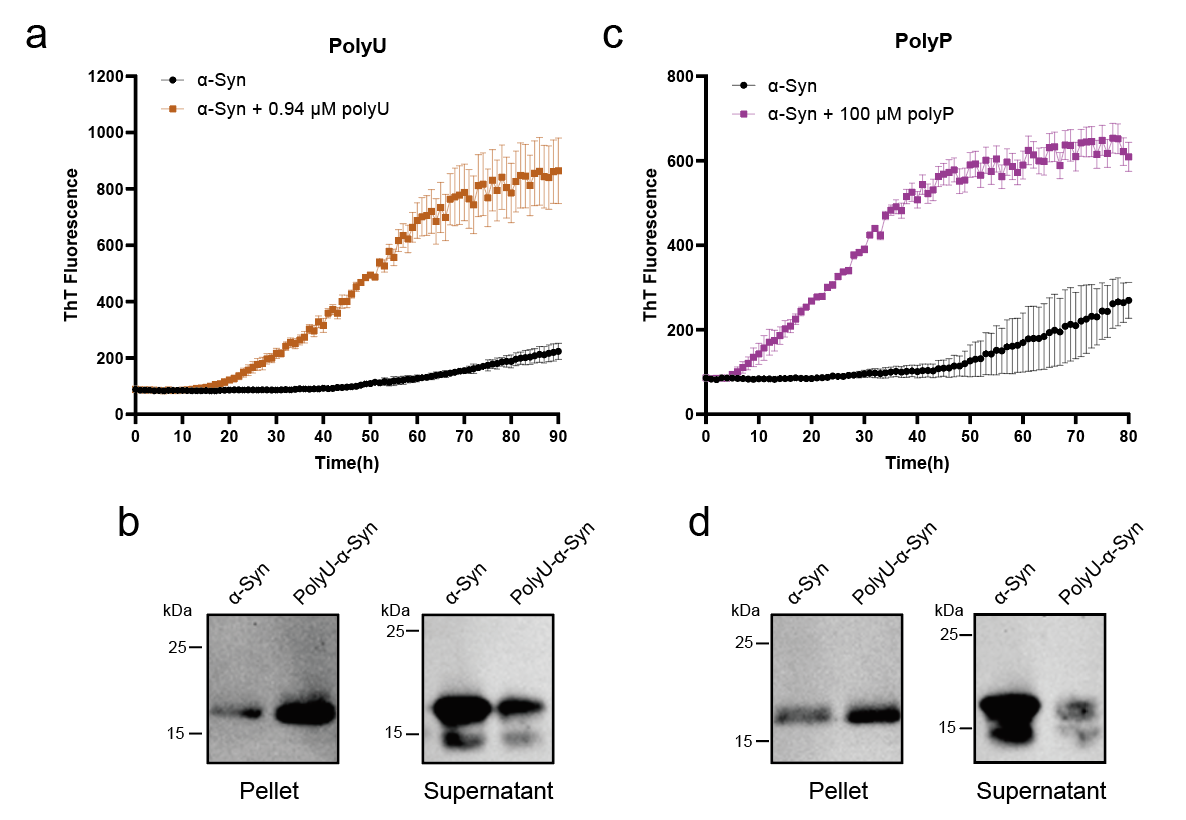


**Fig. S6. ThT kinetics and western blots of polyanion-induced fibrils.**

ThT kinetic curves of α-syn fibril formation in the absence or presence of 0.94 μM polyU (**a**) and 100 μM polyP (**c**). α-Syn monomer concentration is 50 μM. Results are the average of three independent experiments, and the error bars represent SD. Western blots of α-syn from pellet and supernatant from the polyU (**b**) and polyP (**d**) ThT kinetic assays.
