# Supplementary material for: Different charged biopolymers induce α-synuclein to form fibrils with distinct structures
Source: J Biol Chem. 2024 Oct 5;300(11):107862. doi: 10.1016/j.jbc.2024.107862 (PMC11570948; doi:10.1016/j.jbc.2024.107862)
Supplement: Supplementary Tables S1–S3 [file mmc2.docx]

**Table S1.** Kinetic parameters of polyamine-induced α-syn fibrillation in Tris buffer.

| Concentration of polyamines and  α-syn monomer | Group | LogIC50 | Hill Slope ^1^ | Top ^2^ |
| --- | --- | --- | --- | --- |
| 50 μM α-syn (Tris) | 1 | 48.04 | 0.03037 | 1845 |
|  | 2 | 44.76 | 0.02579 | 1831 |
|  | 3 | 46.22 | 0.03189 | 1822 |
| 3.2 μM putrescine: 50 μM α-syn | 1 | 49.06 | 0.04988 | 1860 |
|  | 2 | 51.30 | 0.03940 | 1851 |
|  | 3 | 42.88 | 0.03611 | 1793 |
| 16 μM putrescin: 50 μM α-syn | 1 | 34.86 | 0.03034 | 1792 |
|  | 2 | 31.93 | 0.02744 | 1803 |
|  | 3 | 40.86 | 0.02471 | 1854 |
| 80 μM putrescine: 50 μM α-syn | 1 | 29.68 | 0.02278 | 1944 |
|  | 2 | 28.96 | 0.03180 | 1905 |
|  | 3 | 28.23 | 0.03101 | 2001 |
| 400 μM putrescine: 50 μM α-syn | 1 | 35.21 | 0.01907 | 1989 |
|  | 2 | 35.12 | 0.02570 | 2117 |
|  | 3 | 37.22 | 0.02408 | 2031 |
| 2 mM putrescine: 50 μM α-syn | 1 | 71.56 | 0.02418 | 2184 |
|  | 2 | 53.31 | 0.01353 | 2259 |
|  | 3 | 68.45 | 0.01287 | 2238 |
| 10 mM putrescine: 50 μM α-syn | 1 | 33.51 | 0.01802 | 1090 |
|  | 2 | 42.08 | 0.02458 | 1004 |
|  | 3 | 30.16 | 0.01770 | 1033 |
| 3.2 μM spermidine: 50 μM α-syn | 1 | 42.10 | 0.03180 | 2135 |
|  | 2 | 41.24 | 0.03328 | 2128 |
|  | 3 | 37.50 | 0.02649 | 2162 |
| 16 μM spermidine: 50 μM α-syn | 1 | 36.83 | 0.02472 | 2363 |
|  | 2 | 37.18 | 0.03598 | 2450 |
|  | 3 | 32.40 | 0.02677 | 2269 |
| 80 μM spermidine: 50 μM α-syn | 1 | 44.53 | 0.04088 | 2246 |
|  | 2 | 38.71 | 0.04062 | 2243 |
|  | 3 | 36.67 | 0.03309 | 2130 |
| 400 μM spermidine: 50 μM α-syn | 1 | 43.36 | 0.04548 | 1670 |
|  | 2 | 49.21 | 0.04191 | 1701 |
|  | 3 | 48.22 | 0.02829 | 1690 |
| 2 mM spermidine: 50 μM α-syn | 1 | 59.76 | 0.01652 | 1396 |
|  | 2 | 59.54 | 0.01127 | 1497 |
|  | 3 | 69.34 | 0.01455 | 1467 |
| 10 mM spermidine: 50 μM α-syn | 1 | 23.47 | 0.05834 | 487.5 |
|  | 2 | 23.12 | 0.06186 | 463.9 |
|  | 3 | 19.71 | 0.06460 | 473.9 |

| 3.2 μM spermine: 50 μM α-syn | 1 | 34.88 | 0.03633 | 2310 |
| --- | --- | --- | --- | --- |
|  | 2 | 36.13 | 0.03070 | 2507 |
|  | 3 | 24.26 | 0.02277 | 2464 |
| 16 μM spermine: 50 μM α-syn | 1 | 34.97 | 0.03874 | 2140 |
|  | 2 | 27.94 | 0.03233 | 2062 |
|  | 3 | 29.08 | 0.03316 | 2024 |
| 80 μM spermine: 50 μM α-syn | 1 | 39.54 | 0.03416 | 1678 |
|  | 2 | 34.33 | 0.03562 | 1789 |
|  | 3 | 35.76 | 0.03115 | 1765 |
| 400 μM spermine: 50 μM α-syn | 1 | 33.75 | 0.03320 | 1269 |
|  | 2 | 32.40 | 0.02972 | 1263 |
|  | 3 | 34.37 | 0.03205 | 1175 |
| 2 mM spermine: 50 μM α-syn | 1  2  3 | 23.65  29.29  29.72 | 0.03634  0.04176  0.05266 | 826.0  939.2  840.4 |
| 10 mM spermidine: 50 μM α-syn | 1  2  3 | 12.52  12.13  15.25 | 0.1683  0.1793  0.08392 | 661.6  672.5  641.6 |

^1^ Hill slope: the fibrillation rate.

^2^ Top: the maximum fluorescence intensity (Fmax).

**Table S2.** Kinetic parameters of α-syn fibrillation kinetic curves with or without polyU.

| Concentration of polyU and  α-syn monomer | Group | LogIC50 | Hill Slope | Top |
| --- | --- | --- | --- | --- |
| 50 μM α-syn (Tris-KCl) | 1 | 61.49 | 0.06749 | 932.3 |
|  | 2 | 60.08 | 0.06327 | 869.5 |
|  | 3 | 61.32 | 0.06027 | 760.0 |
| 0.23 μM polyU: 50 μM α-syn | 1 | 47.48 | 0.04134 | 1849 |
|  | 2 | 45.68 | 0.05477 | 1633 |
|  | 3 | 40.47 | 0.04554 | 1750 |
| 0.94 μM polyU: 50 μM α-syn | 1 | 44.28 | 0.1151 | 2512 |
|  | 2 | 34.27 | 0.05468 | 2706 |
|  | 3 | 23.34 | 0.08391 | 2061 |
| 3.75 μM polyU: 50 μM α-syn | 1 | 52.15 | 0.08928 | 2236 |
|  | 2 | 43.00 | 0.06937 | 2280 |
|  | 3 | 50.81 | 0.1321 | 2218 |

**Table S3.** Kinetic parameters of α-syn fibrillation kinetic curves with or without polyP.

| Concentration of polyP and  α-syn monomer | Group | LogIC50 | Hill Slope | Top |
| --- | --- | --- | --- | --- |
| 50 μM α-syn (Tris-KCl) | 1 | 35.52 | 0.06496 | 934.8 |
|  | 2 | 43.16 | 0.05364 | 737.7 |
|  | 3 | 46.66 | 0.04993 | 883.9 |
| 6.25 μM polyP: 50 μM α-syn | 1 | 34.02 | 0.05349 | 1023 |
|  | 2 | 29.13 | 0.1051 | 1002 |
|  | 3 | 37.10 | 0.07578 | 1113 |
| 25 μM polyP: 50 μM α-syn | 1 | 32.47 | 0.07658 | 1119 |
|  | 2 | 28.90 | 0.05023 | 1004 |
|  | 3 | 27.75 | 0.06350 | 1026 |
| 100 μM polyP: 50 μM α-syn | 1 | -121.7 | 0.02884 | 1220 |
|  | 2 | -136.4 | 0.02554 | 1203 |
|  | 3 | -101.0 | 0.03310 | 1148 |
